# Two novel mutations in *ALDH18A1* and *SPG11* genes found by whole-exome sequencing in spastic paraplegia disease patients in Iran

**DOI:** 10.5808/gi.22030

**Published:** 2022-09-30

**Authors:** Sajad Rafiee Komachali, Zakieh Siahpoosh, Mansoor Salehi

**Affiliations:** 1 Cellular, Molecular and Genetics Research Center, Isfahan University of Medical Sciences, Isfahan 8174673461, Iran; 2 Medical Genetics Research Center of Genome, Isfahan University of Medical Sciences, Isfahan 8174673461, Iran; 3 Department of Biology, Faculty of Science, University of Sistan and Baluchestan, Zahedan 9816745845, Iran

**Keywords:** metabolism, mutation, neurological disorder, polyneuropathy, spastic paraplegia, whole-exome sequencing

## Abstract

Hereditary spastic paraplegia is a not common inherited neurological disorder with heterogeneous clinical expressions. *ALDH18A1* (located on 10q24.1) gene-related spastic paraplegias (SPG9A and SPG9B) are rare metabolic disorders caused by dominant and recessive mutations that have been found recently. Autosomal recessive hereditary spastic paraplegia is a common and clinical type of familial spastic paraplegia linked to the *SPG11* locus (locates on 15q21.1). There are different symptoms of spastic paraplegia, such as muscle atrophy, moderate mental retardation, short stature, balance problem, and lower limb weakness. Our first proband involves a 45 years old man and our second proband involves a 20 years old woman both are affected by spastic paraplegia disease. Genomic DNA was extracted from the peripheral blood of the patients, their parents, and their siblings using a filter-based methodology and quantified and used for molecular analysis and sequencing. Sequencing libraries were generated using Agilent SureSelect Human All ExonV7 kit, and the qualified libraries are fed into NovaSeq 6000 Illumina sequencers. Sanger sequencing was performed by an ABI prism 3730 sequencer. Here, for the first time, we report two cases, the first one which contains likely pathogenic NM_002860: c.475C>T: p.R159X mutation of the *ALDH18A1* and the second one has likely pathogenic NM_001160227.2: c.5454dupA: p.Glu1819Argfs Ter11 mutation of the *SPG11* gene and also was identified by the whole-exome sequencing and confirmed by Sanger sequencing. Our aim with this study was to confirm that these two novel variants are direct causes of spastic paraplegia.

## Introduction

In these years, the number of disorders due to changing the pathway of amino acid synthesis has grown so fast. In addition to the number of conditions that has increased, the associated clinical phenotypes have also expanded. All of these are classified as one of advances of next-generation sequencing diagnostics [1].

We all know the fact, that amino acids are the monomers for peptides and proteins. Amino acids play vital roles in intermediate metabolism, and many of them have specific cellular functions of their own. For example, in neurotransmission or energy metabolism and detoxification. From the past until now, the biochemical analysis of levels of amino acids or their degradation products in body fluids has been the indication of diagnosing inborn errors of metabolism [1].

Hereditary spastic paraplegia (HSP) is a not common inherited neurological disorder with heterogeneous clinical expressions. More than 70 different genetic forms of HSP have been found, all types of inheritance such as autosomal dominant (AD), autosomal recessive (AR), X-linked or non-Mendelian mitochondrial maternal transmission have been identified in HSP patients. In this study, we want to report two cases with different mutated genes but both expressing spastic paraplegia phenotypes [2].

*ALDH18A1* gene-related spastic paraplegias (SPG9A [spastic paraplegia-9A] and SPG9B) are rare metabolic disorders caused by dominant and recessive mutations found recently [3]. *ALDH18A1* gene locates on 10q24.1 [4] encodes delta1-pyrroline-5-carboxylate synthetase (P5CS), an enzyme that catalyzes the first and vital step of proline and ornithine biosynthesis from glutamate [5-7]. P5CS is a bi-functional ATP and NADPH-dependent mitochondrial enzyme with gamma-glutamyl kinase and gamma-glutamyl phosphate reductase activities. Its protein catalyzes the reduction of glutamate to delta1-pyrroline-5-carboxylate, a crucial step in the *de novo* biosynthesis of proline, ornithine, and arginine [4,8].

P5CS deficiency includes SPG9A [OMIM #601162], SPG9B [OMIM #616586], ADCL3 [OMIM #616603], and ARCL3A [OMIM #219150]. The P5CS deficiency severity might amplify in the order of SPG9A < SPG9B < ADCL3 ≤ ARCL3A. These forms are found with different levels of P5CS loss of function (LOF) [5]. Disorders of the proline synthesis pathway can cause diseases with a broad range of symptoms from severe natal problems to adult-onset spastic paraplegia [1]. It is good to know that autosomal dominant SPG9A is a neurologic disorder characterized by slowly progressive spasticity, with direct effect on the lower limbs. The age of SPG9A onset usually differs from adolescence to adulthood [7].

Autosomal recessive hereditary spastic paraplegia (ARHSP) is a common and clinical type of familial spastic paraplegia linked to the *SPG11* locus on chromosome 15 in most ARHSP families [9]. *SPG11* is located on 15q21.1 [4] and encodes spatacsin [9]. Loss of spatacsin function due to mutations in the *SPG11* gene inhibits tubule formation in late endosomes/lysosomes, leads to the repletion of cholesterol in this part, due to its disability about exporting out of the organelle. This will result in an actual decrease in the level of plasma membrane cholesterol, leads to disturbing intracellular calcium homeostasis. It is essential to know that loss of spatacsin leads to progressive accumulation of lipids in lysosomes, both in neuronal and non-neuronal cells [10].

There are different symptoms of spastic paraplegia such as short stature, cataracts, nystagmus, gastroesophageal reflux, hiatal hernia, urinary urgency, urinary incontinence, delayed bone age, pes cavus, small carpal bones, lower limb spasticity, lower limb weakness, gait abnormalities, upper motor neuron signs, hypertonia, hyperreflexia, extensor plantar responses, cerebellar signs, motor polyneuropathy, and decreased vibration sense [7,11,12].

In order to distinct pathogenic mechanisms in AD types of spastic paraplegia, more studies are needed to compare the effects of homozygous and heterozygous mutations, especially to find that heterozygous mutations lead to haploinsufficiency or a dominant negative gain-of-function [7].

Although, a lot of causative genes in hereditary spastic paraplegia have been found in these years, there are still about 50% of patients found without genetic reasons, especially in AR types. Rare studies have been done to determine the genetic causes and clinical examination profiles of recessive patients, and there are also additional studies needed in this part [2].

Here, for the first time, we report two cases, the first one which contains NM_002860: c.475C>T: p.R159X mutation of the *ALDH18A1* gene that was identified by whole-exome sequencing and confirmed by Sanger sequencing. The second one contains NM_001160227.2: c.5454dupA: p.Glu1819Argfs Ter11 mutation of the *SPG11* gene and also was identified by whole-exome sequencing and confirmed by Sanger sequencing. Our aim with this study was to propose two novel likely pathogenic variants NM_002860: c.475C>T: p.R159X and NM_001160227.2: c.5454dupA: p.Glu1819Arg fsTer11 can cause spastic paraplegias.

## Methods

### First patient

**Fig. 1.**
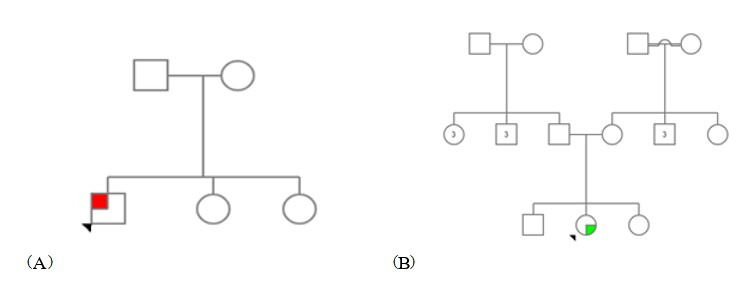
(A) Pedigree of the first proband. (B) Pedigree of the second proband.

Our first proband involves a 45-year-old man that is affected by spastic paraplegias disease and has been diagnosed with muscle atrophy, moderate mental retardation (MR), ehort stature, balance problem, intellectual disability, lower limb spasticity, lower limb weakness, gait abnormalities, upper motor neuron signs, pes cavus, motor polyneuropathy, and decreased vibration sense. It is notable that proband’s maternal family suffered from rheumatis, and his paternal family suffered from asthma; also moderate MR, short stature, cataracts, nystagmus, gastroesophageal reflux, hiatal hernia, delayed bone age, pes cavus, small carpal bones, lower limb spasticity, lower limb weakness, gait abnormalities, upper motor neuron signs, hyperreflexia, cerebellar signs, Parkinson’s disease, seizure, motor polyneuropathy, and decreased vibration sense were reported in his siblings (Fig. 1A).

### Second patient

Our second proband involves a 20-year-old woman that is affected by spastic paraplegia disease and has been diagnosed with progressive muscle weakness, vision problem, lower limb spasticity, lower limb weakness, motor neuropathy, kyphosis, hypotonia, and amyotrophic lateral sclerosis disease symptoms. Notably, there were no signs found in her until she was 12. Her father, mother, 12-year-old sister, and 14-year-old brother show no symptoms until now (Fig. 1B). Local ethics committees received informed consent from both subjected families. Informed consent was obtained from all human adult participants and from the parents or legal guardians of minors in the Genome laboratory of Isfahan. In this study, internal approval has been prepared, adjusted and available in Genome Laboratory of Isfahan. Also, the article has IRCT code = 52793.

### Mutation analysis

Genomic DNA (gDNA) is isolated from the patient’s specimen using a filter-based methodology and quantified. A total of 1.0 μg gDNA per sample was used as input material for the DNA sample preparation. Sequencing libraries were generated using Agilent SureSelect Human All ExonV7 kit (Agilent Technologies, Santa Clara, CA, USA) following the manufacturer’s recommendations, and x index codes added to attribute sequences to sample. Briefly, fragmentation was carried out by the hydrodynamic shearing system (Covaris, Woburn, MA, USA) to generate 180‒280 bp fragments. The Remaining overhangs were converted into blunt ends via exonuclease/polymerase activities, and enzymes were removed. After adenylation of 3′ ends of DNA fragments, adapter oligonucleotides were ligated. DNA fragments with ligated adapter molecules on both ends were selectively enriched in a polymerase chain reaction (PCR) reaction. Captured libraries enriched in a PCR reaction to add index tags to prepare for hybridization. Products were purified using the AMPure XP system (Beckman Coulter, Beverly, MA, USA) and quantified using the Agilent high sensitivity DNA assay on the Agilent Bioanalyzer 2100 system. The qualified libraries are fed into NovaSeq 6000 Illumina sequencers. Then data quality control, analysis and interpretation were run on G9 generation of HP server using UNIX based operating system.

Sanger sequencing was performed by ABI prism 3730 sequencer (Applied Biosystems, Waltham, MA, USA) to validate the pathogenic mutation and segregation the mutation in this family. Mutation Surveyor program version 4.0.9 was used to analyze the sequences (SoftGenetics, State College, PA, USA).

We employed the 48-well thermocycler device (Bio-Rad, Hercules, CA, USA) in this reaction. The materials utilized in PCR, as well as their concentration and amount, were as follows: 6 μL of master (1×), 2 μL of template DNA, 0.5 μL of (10 pmol) forward primer, 0.5 μL of (10 pmol) reverse primer, and 3.5 L of sterile distilled water (total: 12.5 μL). To prepare the PCR solution, we used 0.2 mL microtubes. We poured the mentioned materials into the tubes and stirred them by pipetting. We amplified the noted gene segment with primers.

For the first patient, a 45-year-old man, to amplify the 326-base pair segment, the sequence of forward and reverse primers was CTGAAAGAAATGGTGAGTGCTGCT and GATCTCAAGTAGCAAGTGATGAAGC, respectively. The utilized primers were manufactured by Tag Copenhagen Co. (Kopenhagen, Denmark). The 326-base pair segment was amplified in the thermocycler as follows: cycle 1 for the initial denaturation: once for 5 min at 94°C; cycle 2 including three steps: denaturation, binding the primer to the template strand, and polymerase expansion: 35 times, each for 30 s at 94°C, F: 61.82/R: 59.99°C, and 72°C, respectively; cycle 3 for the final expansion: once for 10 min at 72°C; cycle 4 for maintaining the products: once at 4°C.

For the second patient, a 20-year-old woman, to amplify the 360-base pair segment, the sequence of forward and reverse primers was AGCTTCTTCCTTTTTCTCAACCCAG and TCGCATGTCTCTTTGGATGGAAGG, respectively. The utilized primers were manufactured by Tag Copenhagen Co. The 360-base pair segment was amplified in the thermocycler as follows: cycle 1 for the initial denaturation: once for 5 min at 94°C; cycle 2 including three steps: denaturation, binding the primer to the template strand, and polymerase expansion: 35 times, each for 30 s at 94°C, F: 61.44/R: 62.96°C, and 72°C, respectively; cycle 3 for the final expansion: once for 10 min at 72°C; cycle 4 for maintaining the products: once at 4°C.

## Results

### Proband 1

**Fig. 2.**
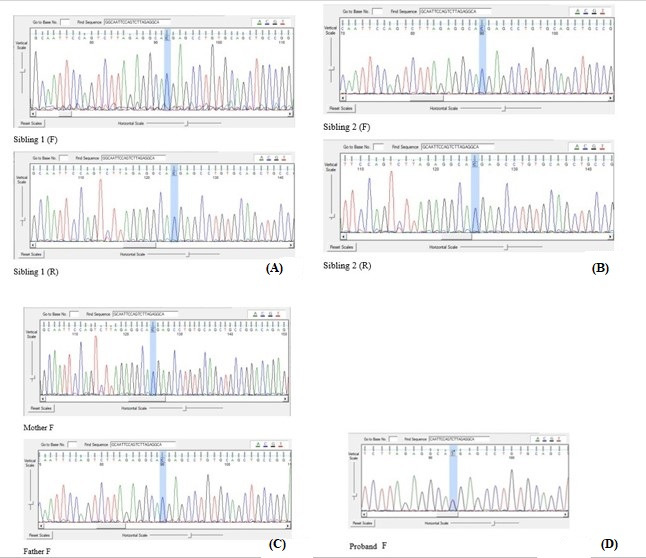
Confirmation sequences of the proband’s sisters, father, mother, and the proband itself. (A) There are both forward strand sequence named sibing 1 (F), and reverse strand sequence named sibing 1 (R). (B) There are both forward strand sequence named sibing 2 (F), and reverse strand sequence named sibing 2 (R). (C) There are proband’s mother forward strand sequence named Mother F, and proband’s father forward strand sequence named Father F. (D) There is proband’s forward strand sequence named Proband F. Sanger sequencing confirmed heterozygosity of the proband 1 in NM_001323412: c.142C>T: p.R159X variant as a novel *de novo* mutation of the *ALDH18A1* gene.

Performing whole-exome sequencing on proband 1, identified a novel heterozygous NM_002860: c.475C>T: p.R159X mutation of the *ALDH18A1* gene located on 10q24.1 and 97396933 position. Sanger sequencing confirmed heterozygosity of NM_002860: c.475C>T: p.R159X mutation in the proband, suggesting it as the putative disease-causing mutation, and AD inheritance pattern in spastic paraplegia 9A disease (Fig. 2).

Notably, this variant is essential if its *de novo* state is confirmed in the patient. Basic clinical information of each analyzed family member are needed for a comprehensive evaluation of the data. In order to Fig. 2, as an evident about his parents, gonadal or germline mosaicism is not found because his sisters are not affected, and also because of the fact that his parents are not affected, the *de novo* state is confirmed about the patient. Based on these findings, additional genetic testing to confirm results, clinical screening tests, or preventive care may be recommended.

There is no report of the NM_002860: c.475C>T: p.R159X mutation in *ALDH18A1* gene in ExAC, 1000G, and other control datasets such as ClinVar. Additionally, this variant does not have a gnomAD exomes entry. c.475C>T: p.R159X [13] DANN score [14] was 0.9974, and was found as pathogenic mutation in EIGEN predictor [15]. This variant was found as a damaging variant in FATHMM-MKL [16], BayesDel noAF [17], and BayesDel addAF [17] meta-predictors through searching dbNSFP v4 [18]. Also, it is notable that NM_002860: c.475C>T: p.R159X mutation was found as a disease-causing mutation in mutation taster predictor [19]. In PHRED database, NM_002860: c.475C>T: p.R159X raw score was reported as 6.936331, and its PHRED score was reported 36, as we know in 97396933 position [20,21].

Due to American College of Medical Genetics and Genomics (ACMG) classification, NM_002860: c.475C>T: p.R159X variant is likely pathogenic. By studying PVS1 rule, it was found that Null variant (nonsense) in gene *ALDH18A1*, predicted to cause nonsense-mediated decay (NMD). Loss-of-function is a known mechanism of disease (gene has 15 pathogenic LOF variants and gnomAD Loss-of-Function Observed/Expected = 0.326 is less than 0.755), associated with Cutis laxa, autosomal recessive, type IIIA, *ALDH18A1*-related de Barsy syndrome, autosomal dominant complex spastic paraplegia type 9B, autosomal recessive complex spastic paraplegia type 9B, Cutis Laxa, autosomal dominant 1 and 4 more. The truncated region affects 1 functional domain: UniProt protein P5CS_HUMAN region of interest 'Glutamate 5-kinase'. The truncated region contains nine pathogenic LOF variants. Additionally, by studying PM2 rule it was found that GnomAD exomes homozygous allele count = 0 is less than 2 for AD/AR with good gnomAD exomes coverage = 85.0. Variant not found in gnomAD genomes with good gnomAD genomes coverage = 31.6 [13].

### Proband 2

**Fig. 3.**
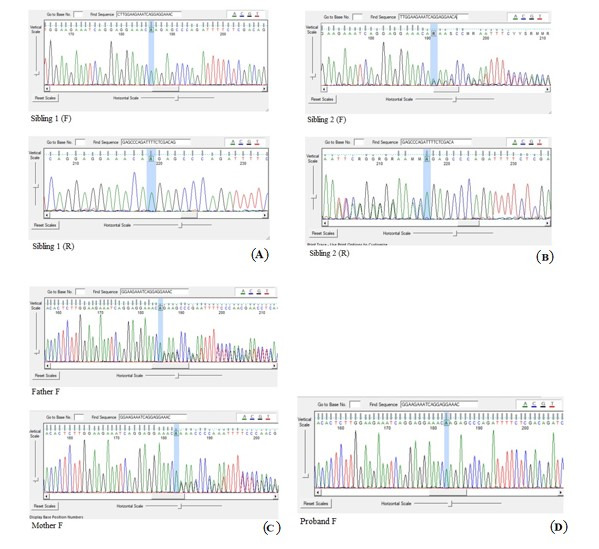
Confirmation sequences of the proband’s sisters, father, mother, and the proband itself. (A) There are both forward strand sequence named sibing 1 (F), and reverse strand sequence named sibing 1 (R). (B) There are both forward strand sequence named sibing 2 (F), and reverse strand sequence named sibing 2 (R). (C) There are proband’s father forward strand sequence named Father F, and proband’s mother forward strand sequence named Mother F. (D) There is proband’s forward strand sequence named Proband F. Sanger sequencing confirmed homozygosity of the proband 2, homozygosity of her sister (A), heterozygosity of her brother (B) and heterozygosity of her father and mother (C) in NM_001160227.2: c.5454dupA: p.Glu1819Argfs Ter11 mutation of the *SPG11* gene.

Performing whole-exome sequencing on proband 2, identified a novel homozygous NM_001160227.2: c.5454dupA: p.Glu1819Argfs Ter11 mutation of the *SPG11* gene located on 15q21.1 and 44584226 position. Sanger sequencing confirmed homozygosity of NM_001160227.2: c.5454dupA: p.Glu1819Argfs Ter11 mutation in the proband, suggesting it as the likely pathogen disease-causing mutation, and AR inheritance pattern in spastic paraplegia 11 disease (Fig. 3). The proband showed no symptoms until she was 12, and now, when her sister is homozygous for this likely pathogen variant and at the same age, it is logical that the age of spastic paraplegia 11 disease is delayed in her.

Basic clinical information of each analyzed family member are needed for a comprehensive evaluation of the data. In order to Fig. 3, by Sanger confirming, it is found that her father, mother, and her brother are affected as heterozygous for *SPG11*, and her sister is affected as homozygous for *SPG11*, so it is confirmed that NM_001160227.2: c.5454dupA: p.Glu1819Argfs Ter11 variant is an ARHSP cause. Based on these findings, additional genetic testing to confirm results, clinical screening tests, or preventive care may be recommended.

No data is available for NM_001160227.2: c.5454dupA: p.Glu1819Argfs Ter11 mutation in *SPG11* gene in ExAC, 1000G, and other control datasets such as ClinVar. Additionally, this variant does not have a gnomAD exomes entry and there are no data available in DANN, CADD, dbNSFP, and dbscSNV (c.5454dupA: p.Glu1819Argfs Ter11) [13]. Through IlluQC level, PHRED score cut off was 20, and in PHRED database, NM_001160227.2: c.5454dupA: p.Glu1819Argfs Ter11 score was reported as 36 as we know in 44584226 position [20,21].

Due to ACMG classification, NM_001160227.2: c.5454dupA: p.Glu1819Argfs Ter11 variant is likely pathogenic. By studying PVS1 rule, it was found that null variant (nonsense) in gene *ALDH18A1*, predicted to cause NMD. Loss-of-function is a known mechanism of disease (gene has 15 pathogenic LOF variants and gnomAD Loss-of-Function Observed/Expected = 0.326 is less than 0.755), associated with Cutis laxa, autosomal recessive, type IIIA, *ALDH18A1*-related de Barsy syndrome, autosomal dominant complex spastic paraplegia type 9B, autosomal recessive complex spastic paraplegia type 9B, Cutis Laxa, autosomal dominant 1 and 4 more. The truncated region affects 1 functional domain: UniProt protein P5CS_HUMAN region of interest 'Glutamate 5-kinase'. The truncated region contains nine pathogenic LOF variants. Additionally, by studying PM2 rule it was found that GnomAD exomes homozygous allele count = 0 is less than 2 for AD/AR with good gnomAD exomes coverage = 85.0. Variant not found in gnomAD genomes with good gnomAD genomes coverage = 31.6 [13].

### Mutated protein models

**Fig. 4.**
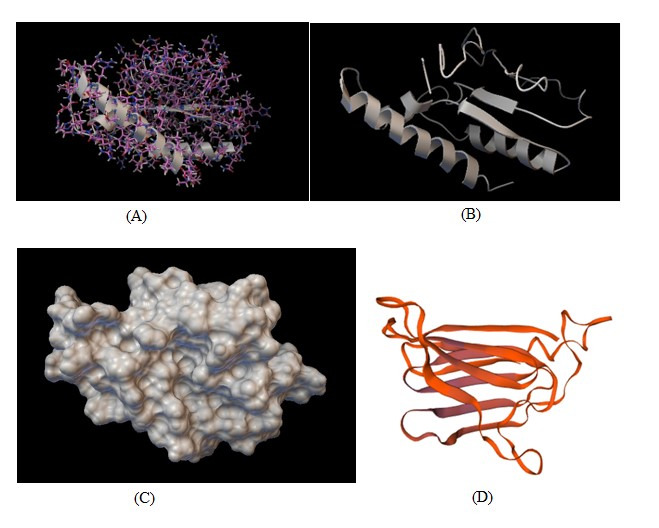
Mutated truncated protein models of the first proband are visible in three forms: balls and sticks (A), ribbon (B), and molecular surface (C), and the second proband protein model is shown in ribbon form (D). Models A, B, and C were created template-free by web based I-tasser software using protein sequence from Protein Data Bank [23], and model D, the mutated truncated protein model was made template free by web based SWISS-MODEL software using protein sequence from Protein Data Bank [24].

Eliminating the N- or C-terminal of a protein by proteolysis or manipulation of the structural gene, or premature termination of protein elongation is due to the presence of a stop codon in its structural gene as a result of a nonsense mutation will result in a truncated protein [22]. As found in our first proband, p.R159X mutation will result in a truncated protein, and it is obvious why P5CS cannot do its role as well as before after mutation. In our second proband, p.Glu1819Argfs Ter11 mutation will also result in a truncated protein. So *SPG11* protein cannot do its role completely as before mutation (Fig. 4).

## Discussion

Based on the results, our first proband that is heterozygous for NM_001323412: c.142C>T: p.R159X variant as a novel mutation of the *ALDH18A1* gene, is a likely pathogenic variant that has a significant risk factor for spastic paraplegia 9A disease. Neither his sisters nor his parents are affected, so it is confirmed that NM_001323412: c.142C>T: p.R159X variant is a *de novo* one.

Our second proband that is homozygous for NM_001160227.2: c.5454dupA: p.Glu1819Argfs Ter11 variant as a novel mutation of the *SPG11* gene is a likely pathogenic variant that has a significant risk factor for spastic paraplegia 11 disease. By Sanger confirming, it is found that her father, mother, and her brother are heterozygous for *SPG11* mutation, and her sister is homozygous for *SPG11* mutation, so it is confirmed that NM_001160227.2: c.5454dupA: p.Glu1819Argfs Ter11 variant is an ARSHP cause.

Over the past decade, there have been remarkable advances in the identification of the genes responsible for spastic paraplegia and the understanding of the molecular pathogenesis of these conditions. Notably, the location of the mutations in the gene has an influence on syndrome [25]. To date, several pathogenetic mechanisms have been identified as responsible for spastic paraplegia, including oxidative stress, dysfunction of axonal development and axonal transport, abnormal lipid metabolism, altered DNA repair, dysmyelination, disrupted autophagy, abnormal cell signaling, and abnormal membrane trafficking [26].

Currently, no specific therapies are found to prevent, delay, or reverse the progressive disability in spastic paraplegia. Treatment is exclusively symptomatic, and its aims are to reduce muscle spasticity and urinary urgency, and improve strength and gait. Therapeutic options include physical therapy, oral antispastic drugs (baclofen, progabide, dalfampridine), botulinum toxin therapy, and surgical baclofen pump implantation. However, there are no guidelines or recommendations from clinicians for patients about selecting the most suitable treatment and finding the best therapies [26].

There is no gene-specific therapy developed for spastic paraplegia thus far, but in another strategy, physiopathological studies in animal models and neurons derived from their induced pluripotent stem cells have provided acceptable therapeutic targets for some forms of spastic paraplegia. Physiopathological studies can lead to the recognition of therapeutic approaches for different forms of spastic paraplegia. However, in the future, the challenge will be to develop a particular treatment for each spastic paraplegia subtype, given the considerable heterogeneity of these diseases. Rare diseases deserve specific trials to develop new treatment strategies, mainly with homogeneous cohorts. In the way of finding new treatment strategies, there is a lack of natural history data, especially longitudinal biomarker analysis, obviously [27].

As a new method, mRNA-based protein supplementation is a powerful tool to reward for the lack of proteins in monogenetic disorders caused by loss-of-function mutations. It offers a potentially curative treatment option, especially in those diseases where the protein is expressed predominantly in organs that can be reached by intravenous delivery. Here, supplementation of mRNA could rehabilitate physiological conditions and may provide a potential novel therapeutic strategy [28].

To our knowledge this is the first report of likely pathogenic NM_001323412: c.142C>T: p.R159X, and NM_001160227.2: c.5454dupA: p.Glu1819Argfs Ter11 variants discovery. Consequently, the results of the present study may be of importance in genetic counseling.
